# Maintenance suppression reduces the accessibility of visual information in working memory regardless of its normative valence

**DOI:** 10.3389/fcogn.2024.1487851

**Published:** 2024-11-07

**Authors:** Caleb N. Jerinic-Brodeur, Marie T. Banich, Jarrod A. Lewis-Peacock

**Affiliations:** ^1^Department of Psychology, University of Texas at Austin, Austin, TX, United States; ^2^Institute of Cognitive Science, University of Colorado, Boulder, CO, United States; ^3^Department of Psychology and Neuroscience, University of Colorado, Boulder, CO, United States

**Keywords:** working memory, suppression, removal, emotion, cognitive control, inhibition

## Abstract

Intentional removal of unwanted information allows us to focus on our current goals. Previous research has shown that suppressing the maintenance of neutral images in working memory can impair access to that information in immediate and delayed memory tests. However, it remains unclear whether maintenance suppression has the same impact on emotionally valenced images. Intrusive thinking (e.g., rumination) often involves negative thoughts that persist as individuals attempt to push them out of mind. Given the emotional nature of intrusive information that can repeatedly enter working memory, it is important to understand how the valence of information affects the ability to remove it. Participants in a non-clinical sample completed a working memory removal experiment using group-normed images with positive and negative valence. Participants encoded two images of the same valence on each trial, were cued to suppress or maintain one of them during a brief delay period, and then responded to a memory probe in which they indicated whether the test image had been presented on the current trial, regardless of whether or how it was cued. Our results demonstrate that participants were faster, relative to uncued items, to endorse an item that had been cued for maintenance, and slower to endorse an item that had been cued for suppression. Importantly, this pattern held for both positive and negative items and did not differ between valences. These findings replicate those obtained using emotionally neutral stimuli. Thus, this study demonstrates that maintenance suppression reduces the accessibility of visual information in working memory, regardless of its emotional valence, and suggests that this cognitive strategy could potentially be an effective tool in reducing intrusive thoughts that occupy the focus of attention.

## Introduction

The ability to control the contents of our current thoughts is a vital function of human cognition, which is supported by working memory. Working memory allows individuals to flexibly guide goal-directed behavior by providing efficient access to useful, task-relevant information. Due to its capacity limits (Cowan, 2001; Luck and Vogel, 2013), removing irrelevant or unwanted information from working memory allows for its efficient use (Lewis-Peacock et al., 2018). Many psychiatric disorders are accompanied by deficits in removing information from working memory (Foland-Ross et al., 2013; Joormann and Gotlib, 2008; Wegner and Zanakos, 1994), but difficulty controlling intrusive thoughts is also a common occurrence in non-clinical populations (Bywaters et al., 2004; Newby and Moulds, 2011).

Previous research has proposed that individuals can utilize cognitive control strategies to remove information from working memory (Banich et al., 2015; Kim et al., 2020; Lewis-Peacock et al., 2018). This work has identified three distinct strategies to remove a thought from working memory: suppress that specific thought, replace that thought with another thought, or clear the mind of all thoughts. Suppression is the only removal method of these that reduces access to the removed information, as indexed by slowed response times to the suppressed item on a short-term recognition memory test as compared to an item that has been maintained (Kim et al., 2020). These results suggest that suppression can overcome the enhanced access typically afforded to information that is actively maintained in working memory. This interpretation was further supported by the observation that suppressing an item from working memory eliminated its proactive interference on subsequent encoding of an item of the same category, whereas replacing an item or clearing all items in working memory did not. Classification analyses revealed that each of these cognitive control strategies are supported by unique patterns of brain activity and differentially alter the encoded information, at distinct timescales. These results suggest that while these processes manipulate information in unique ways, information in working memory is only successfully removed through suppression. We refer to this suppression process acting on information in working memory as “maintenance suppression” to differentiate it from “retrieval suppression” which has been studied extensively and involves the inhibition of retrieval of cued associates from long-term memory (for review, see Anderson and Hanslmayr, 2014).

Decades of research have been committed to determining the factors that lead to enhanced memory. One modulatory factor that has been extensively studied is emotion, and the general consensus from this line of research is that emotional (specifically negative) information is prioritized by human long-term memory systems (for review, see LaBar and Cabeza, 2006). Researchers have also assessed how emotion influences the ability to control information in long-term memory, specifically in directed forgetting and retrieval suppression (i.e., Think/No-Think) paradigms. Studies investigating the role of emotion in directed forgetting have found that emotional information is typically harder to forget compared to neutral information. However, this effect varies across studies and is likely driven by differences in arousal (for meta-analysis, see Hall et al., 2021). Research investigating the role of emotion in retrieval suppression has demonstrated that both emotional and neutral information can be forgotten under direct suppression instructions (van Schie et al., 2013), and that these inhibitory effects may even be increased when suppressing emotional memories as compared to neutral ones (Depue et al., 2006).

While a large portion of research focuses on the impacts of emotion on episodic long-term memory, there have also been numerous studies focused on the role of emotion in working memory (Mikels and Reuter-Lorenz, 2019). While emotion provides a robust enhancement to long-term memories, there are not consistent effects on working memory performance. Individuals have shown better recall for negative information compared to neutral information after 24–48 h in incidental encoding paradigms, but with no differences in immediate working memory performance. Specifically, *n*-back accuracy is the same for emotional and neutral information, but updating performance was hindered (i.e., slowed reaction times) in the presence of emotional information (Kensinger and Corkin, 2003). Further research investigating the effects of emotion on working memory have found mixed results. Some studies find that emotion enhances working memory performance, while others find decrements in performance. This disconnect is likely a result of the type of emotional stimuli utilized, as well as the underlying processes involved in the various working memory paradigms (for meta-analysis, see Ribeiro et al., 2019). Despite these large bodies of work, it is unknown how emotion influences the ability to intentionally control the contents of working memory through maintenance suppression.

The current study aimed to examine if maintenance suppression can impair access to emotional information in working memory. We employed a working memory removal paradigm similar to our prior work (Kim et al., 2020). We manipulated emotion by instructing participants to encode pairs of images, which on some trials were both negative and on other trials were both positive. Encoding was followed by a cue to maintain or suppress the maintenance of one of the preceding images. Each trial ended with a recognition memory probe to assess both response time and accuracy. We hypothesized that suppressing negative information from working memory should be more difficult compared to positive information because of attentional capture caused by its increased salience (Anderson and Phelps, 2001; Bargh et al., 1992; Pratto and John, 1991; Riemann and McNally, 1995; Williams et al., 1996). We also hypothesized that there would be no significant differences in the ability to maintain negative vs. positive information in working memory. Additionally, we did not expect to observe any effects of valence on recognition accuracy because memory was probed immediately at the end of each trial.

## Methods

### Participants

The experimental procedure was approved by the University of Texas at Austin's Institutional Review Board (IRB protocol #2013-10-0110). A total of 105 participants (24 male; age, *M* = 19.34, SD = 2.88) were recruited from the University of Texas at Austin and the Austin, TX area and completed the experiment in-person. All participants had normal or corrected-to-normal vision, were fluent English speakers, provided informed consent, and received either course credit or monetary compensation ($12/h). Seventeen participants were excluded from the final analyses based on our exclusion criteria used in prior research (recognition memory accuracy < 75%; Kim et al., 2020). The remaining 88 participants (18 male; age, *M* = 19.12, SD = 1.12) were included in all analyses.

Our prior (unpublished) behavioral studies examining within-subject contrasts yields effect sizes of 0.43 for the contrast between conditions (i.e., maintain vs. suppress) and 0.3 for the manipulated vs. non-manipulated item within condition. G^*^Power 3.1 (Faul et al., 2009) indicates that Ns = 45–80 are required to achieve 80% power at *p* < 0.05. We collected data from ~100 participants to reach the required N after exclusions.

### Stimuli

The experiment was designed using PsychoPy (Peirce et al., 2019) and Python3. All stimuli were presented on a black background with white text and fixation points. Stimuli consisted of colored scene images rated for emotional valence and arousal. These images were compiled from multiple existing open access datasets of emotional images (e.g., IAPS: Lang et al., 2008; NAPS: Marchewka et al., 2014; OASIS: Kurdi et al., 2017). Because these images were previously rated using independent subject pools and different rating scales, we collected a new common set of ratings for the image set used in the current study using Amazon's Mechanical Turk (MTurk). Participants provided valence and arousal ratings for each image. All ratings ranged from −3 (Valence: “extremely negative”; Arousal: “very relaxed/calm”) to 3 (Valence: “extremely positive”; Arousal: “very excited/aroused”), with 0 being classified as “neutral.” Ratings were collected for 726 images by 188 individuals, with ~21 unique ratings per image.

**Figure 1:**
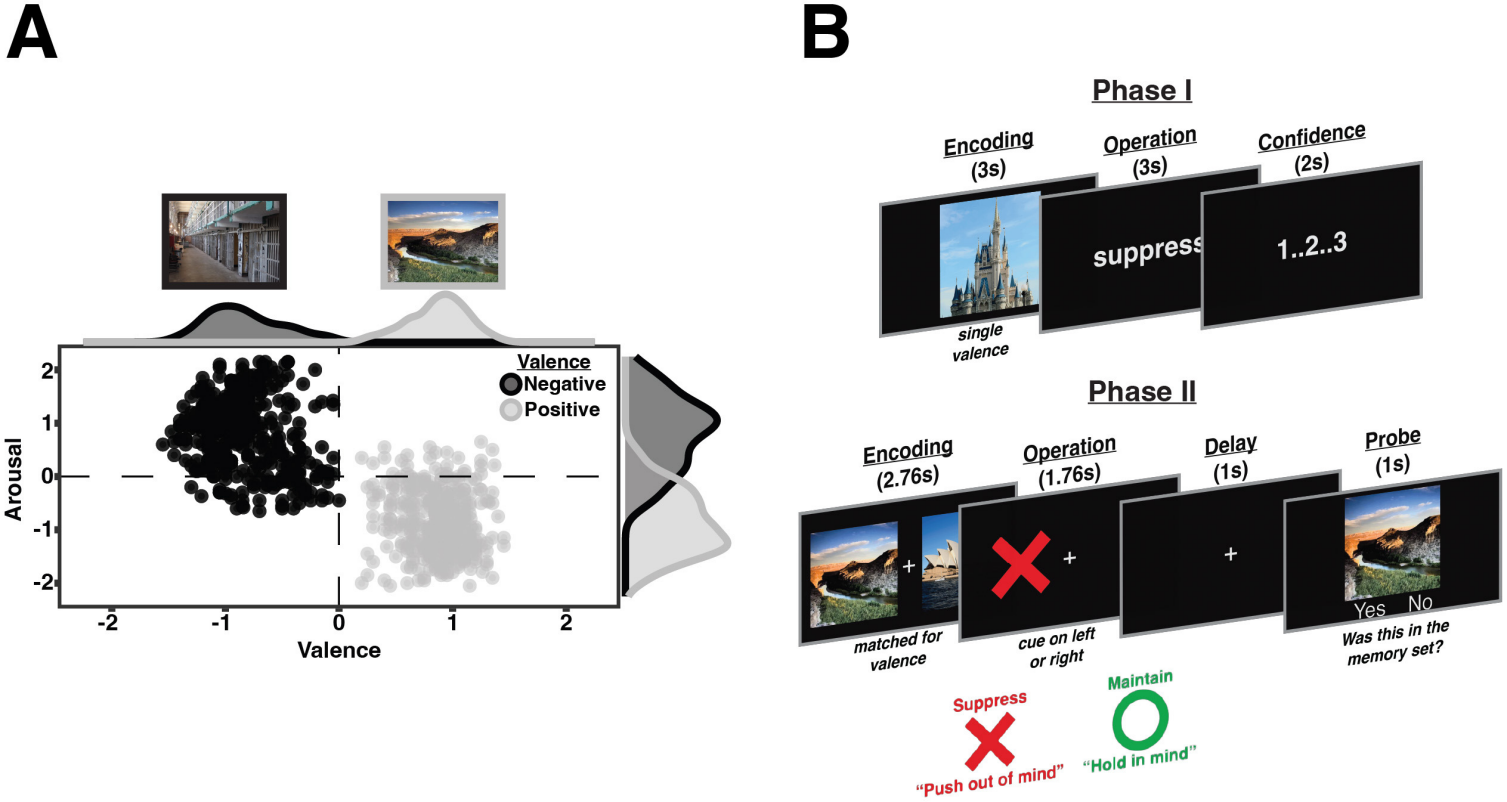
Normative stimulus ratings and behavioral paradigm. **(A)** Distribution of the normative ratings for the images used in the current study plotted as a circumplex. The horizontal dashed line separates high and low arousal images, while the vertical dashed line separates positive and negative images. Black points represent negative images and gray points represent positive images. **(B)** Two-phase working memory removal task. In Phase I, participants practice suppressing images of their group assigned valence. In Phase II, valence is interleaved, and participants make recognition judgments after being cued to suppress (red X) or maintain (green circle) one of the memory items. If the probe stimulus matched one of the memory items on that trial, they were to press “Yes” even if it had been cued for suppression.

Group-normative averages for valence and arousal ratings are visualized in Figure 1A. Images with a mean valence rating < 0 were categorized as negative images (*N* = 366). Images with a mean valence rating >0 were categorized as positive images (*N* = 360). Images with a mean arousal rating < 0 were categorized as low arousal images (*N* = 377). Images with a mean arousal rating >0 were categorized as high arousal images (*N* = 349). Negative stimuli were largely rated as highly arousing (*N* = 317), while positive images were largely rated as lowly arousing (*N* = 328). Stimuli were randomly selected for each participant from this full set.

### Procedure

We designed a mixed within- and between-subjects experiment to address our hypotheses. Participants completed a two-phase working memory removal task adapted from our prior study (Kim et al., 2020). Participants were randomly assigned to one of two groups prior to beginning the experiment: “Practice Positive” (*N* = 44) and “Practice Negative” (*N* = 44). We wanted to explore if practicing to suppress one valence of information generalizes when later encountering both valences of information. This manipulation was included to mirror the design of a planned neuroimaging study but as discussed below, did not influence the results. Because there were no group-based differences in performance, the data was collapsed across groups for all analyses.

During Phase I (Figure 1B, top), participants practiced two cognitive control operations on images of their group-assigned valence (e.g., the “Practice Positive” group was only exposed to positively valenced images). Phase I consisted of eight blocks of 20 trials, with a 60 s break following each block. Within each block, participants performed 10 trials of each operation (i.e., maintain and suppress). Importantly, operation order was pseudo-randomized such that no more than three trials in a row required the same operation. Before the experiment began, participants were briefed on the cognitive control operations and how they should manipulate information in each manner.

For the maintain operation, participants were instructed to deliberately continue to think of the image that preceded the cue. For the suppress operation, participants were instructed to push the image that preceded the cue out of their mind, as they would suppress a cough. More specifically, every time that image bubbled back into their mind, they should continue to attempt to push it out. Importantly, participants were told during the suppress operation they should not be thinking about anything else or trying to clear their mind completely.

Each trial began with a single image presented in the center of the screen for participants to encode for 3 s. An instruction screen then appeared for 3 s with an operation cue which indicated how participants should manipulate the encoded image. The operation cue was the written name of the operation (i.e., maintain or suppress). After a 3 s delay, participants were asked to make a subjective confidence rating within 2 s. Participants were instructed to rate how well they believed they performed the given cognitive control operation in-line with the experimenter's instructions-−1: “Lousy,” 2: “Decent,” 3: “Nailed it!” Each trial ended with a randomly jittered central fixation cross for 1–2 s. This subjective rating was included to mirror the trial timing of a planned neuroimaging study. There was no significant relationship between these subjective ratings and later performance, so this data will not be discussed further.

Completion of Phase I was followed by a 5-min break prior to beginning Phase II (Figure 1B, bottom). While image valence was manipulated between-subjects in Phase I, valence was interleaved in Phase II. Phase II consisted of six blocks of 16 trials, with a 60 s break after the third block. Within each block, participants performed eight trials of each operation (i.e., maintain and suppress) and eight trials consisted of images of each valence. Importantly, operation order and valence order were pseudo-randomized so there were no more than three trials with the same operation or the same valence in a row. Each trial started with a 2.76 s encoding period where participants were shown two target images, with one to each side of a central fixation cross. Participants were instructed to encode both images, as they were unaware which image would be later manipulated. Importantly, the two target images encoded on a given trial were of the same valence. Additionally, the two images were matched for luminance to ensure contrast between images was not driving memory performance. The luminance value of each image was obtained and used to sort the images into four different bins through a quartile split. Once these bins were created, we calculated the mean luminance within each of the four bins. Each image's luminance was then adjusted to the mean value of its respective bin. This method was performed separately for negative and positive images. Next, an instruction screen appeared for 1.76 s with an operation cue on one side of the central fixation cross. The location of the operation cue indicated which target item should be manipulated with the given operation. This item is classified as the “Manipulated” item, while the un-cued item is classified as the “Non-manipulated” item. The cues were presented as colored shapes, and participants were debriefed on their associations to the operations prior to the start of Phase II. A green “O” cued that an item was to be maintained, while a red “X” cued that an item was to be suppressed. After a brief delay (1 s), a recognition probe was presented in the center of the screen for 1 s and participants were required to make a response within 2.5 s. Participants responded as to whether the probe item had been presented at the beginning of the current trial, regardless of whether the item was manipulated or not. Participants responded with the “F” key (indicating a “Yes” response) if the probe image was encoded on the current trial and the “J” key (indicating a “No” response) if the probe image was not encoded on the current trial. Half of the recognition probes consisted of a target item (“Valid probes”; manipulated or non-manipulated item) and required a “Yes” response, while the other half consisted of novel images (“Negative probe”) and required a “No” response. Each trial ended with a 1.5 s blank inter-trial interval.

### Analyses

Since no memory responses were made during Phase I of the experiment, the following exclusions and analyses were performed on the data from Phase II. Participants (*N* = 17) with recognition accuracy < 75% on maintain or suppress trials were excluded from all analyses to ensure we did not analyze data from participants who may have been confused about the task. Reaction times to the memory probes were calculated for positive probe trials (i.e., trials that required a “Yes” response). Only correct positive probe trials were included in the final analyses. Individual trials were excluded for each participant if the reaction time was below 200 ms or >2.5 standard deviations of the within-subject mean reaction time (*M* = 5.10%, SEM = 0.35%).

Participant means were calculated for accuracy and reaction times to recognition memory probes. We conducted two repeated measures analysis of variances (ANOVA) to determine if there was an effect of practice group on the memory measures; one for accuracy and another for reaction times. Data was then collapsed across groups (see **Results**). One-way within-subjects ANOVAs were used to compare differences on both memory measures for each valence-operation (e.g., negative-suppress) pair; one for manipulated items and another for non-manipulated items. The collapsed data was then submitted to two separate repeated measures ANOVAs to test for effects of valence; one for accuracy and another for reaction times. Paired sample *t*-tests were applied separately to both memory measures to compare differences for manipulated vs. non-manipulated items for each valence-operation pair. Bayes factors were calculated for all behavioral analyses. No corrections were applied to any of the statistical tests. The data was analyzed in R; Bayes factors were calculated using the BayesFactor package and the results were visualized with the ggplot2 package (Wickham, 2016).

## Results

### Normative emotion ratings

Descriptive statistics were calculated for the normative emotion ratings for the stimuli that were collected on a separate sample of participants via MTurk. Zero represents a “neutral” rating. Images that were classified as being negative (*N* = 366) had a mean valence rating of −0.83 (SD = 0.33) and a mean arousal rating of 0.80 (SD = 0.68). Images that were classified as being positive (*N* = 360) had a mean valence rating of 0.87 (SD = 0.25) and a mean arousal rating of −0.86 (SD = 0.59).

To further confirm these qualitative classifications, we conducted a one-sample *t*-tests to determine if these ratings significantly differed from a neutral rating of zero. The *t*-tests revealed that the mean valence rating for negative images was significantly below zero [*t*_(365)_ = −48.54, *p* < 0.001, *d* = 2.54], while the mean valence rating for positive images was significantly above zero [*t*_(359)_ = 65.26, *p* < 0.001, *d* = 3.44]. The mean arousal rating for negatively valenced images was significantly above zero [*t*_(365)_ = 22.64, *p* < 0.001, *d* = 1.18], while the mean arousal rating for positively valenced images was significantly below zero [*t*_(359)_ = −27.61, *p* < 0.001, *d* = 1.45].

We then applied a Welch's two-sample *t*-test to compare whether valence or arousal significantly differed between items classified as negative vs. positive. These *t*-tests revealed that both valence [*t*_(685.5)_ = −78.39, *p* < 0.001, *d* = 5.81] and arousal [*t*_(713.5)_ = 35.23, *p* < 0.001, *d* = 2.61] significantly differed between images classified as negative compared to images classified as positive.

Because stimuli were randomly selected for each participant from the full set, we wanted to ensure there were no significant differences in the valence or arousal of stimuli used for each participant that could potentially influence our results. Participant was treated as a fixed effect in a linear model to quantify any differences in valence and arousal across individuals. There was no significant effect of participant on image valence [*t*_(9, 520)_ = 0.01, *p* = 0.99] or arousal [*t*_(9, 520)_ = 0.54, *p* = 0.59].

### Group differences

No significant effects of the assigned practice group in Phase I were observed on memory responses during Phase II [Accuracy: *F*_(1, 86)_ = 0.40, *p* = 0.53, $\eta_{p}^{2}$ = 0.005, BF = 0.13, *f*^2^ = 0.15; Reaction Time: *F*_(1, 86)_ = 0.60, *p* = 0.44, $\eta_{p}^{2}$ = 0.007, BF = 0.31, *f*^2^ = 0.45]. As such, the final analyses have been collapsed across groups.

### Recognition memory accuracy

**Figure 2:**
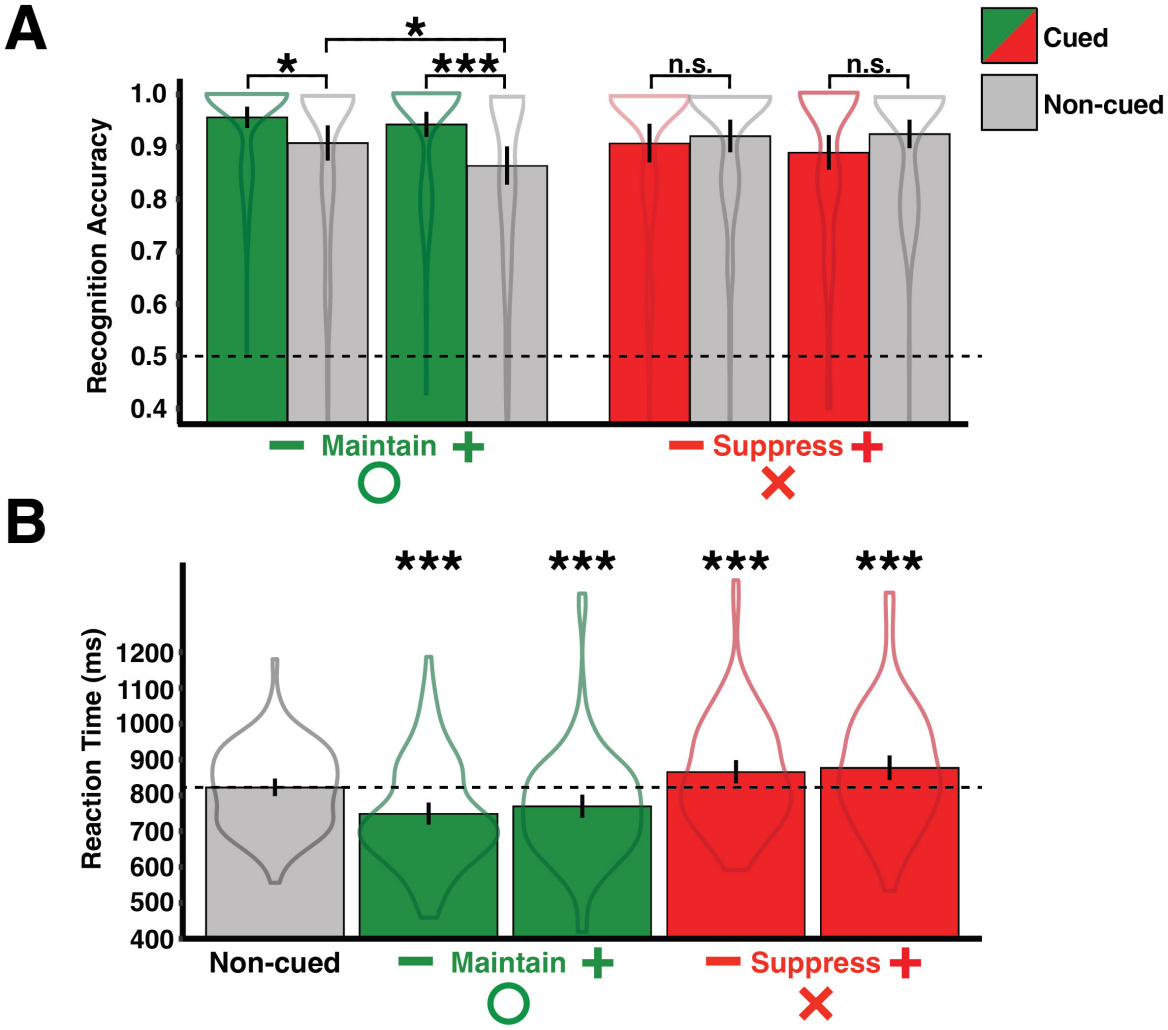
Behavioral memory results. **(A)** Accuracy means shown on the *y*-axis as a function of working memory operation, image valence, and probe status (dashed line represents chance performance). **(B)** Reaction time (ms) means shown on the *y*-axis as a function of working memory operation and image valence, compared to a common baseline (represented by the dashed line). *p < 0.05, ***p < 0.001; uncorrected.

Mean accuracy was calculated for manipulated and non-manipulated items for each valence-operation (e.g., negative-suppress) pair (Figure 2A). There was a significant difference in accuracy for manipulated items across valence-operation pairs [*F*_(3, 348)_ = 4.45, *p* < 0.01, $\eta_{p}^{2}$ = 0.04; BF = 4.21, *f*^2^ = 1.31], as well as a significant difference in accuracy for non-manipulated items across all valence-operation pairs [*F*_(3, 348)_ = 2.92, *p* < 0.05, $\eta_{p}^{2}$ = 0.02; BF = 0.57, *f*^2^ = 1.30]. Maintain cues improved accuracy for both negative [*t*_(87)_ = 2.60, *p* < 0.05, *d* = 0.37; BF = 2.76, *r* = 0.71] and positive [*t*_(87)_ = 3.60, *p* < 0.001, *d* = 0.54; BF = 41.10, *r* = 0.71] items, compared to non-manipulated items. Suppress cues did not affect accuracy for either negative [*t*_(87)_ = −0.59, *p* = 0.56, *d* = 0.09; BF = 0.14, *r* = 0.71] or positive [*t*_(87)_ = −1.62, *p* = 0.11, *d* = 0.25; BF = 0.41, *r* = 0.71] items, compared to non-manipulated items. There was no impact of valence on accuracy during Suppress trials [*F*_(1, 87)_ = 0.36, *p* = 0.55, $\eta_{p}^{2}$ = 0.004; BF = 0.13, *f*^2^ = 0.15], however there was an effect of valence on accuracy during Maintain trials [*F*_(1, 87)_ = 4.95, *p* < 0.05, $\eta_{p}^{2}$ = 0.05; BF = 0.64, *f*^2^ = 1.76]. The valence effect during Maintain trials was driven by decreased accuracy to positive non-manipulated items compared to negative non-manipulated items [*t*_(87)_ = −2.29, *p* < 0.05, *d* = 0.26, BF = 1.40, *r* = 0.71].

### Recognition memory reaction time

Mean reactions times were calculated for manipulated and non-manipulated items for each valence-operation (e.g., negative-suppress) pair (Figure 2B). There was a significant difference in reactions times for manipulated items across valence-operation pairs [*F*_(3, 348)_ = 16.04, *p* < 0.001, $\eta_{p}^{2}$ = 0.12; BF = 11,628,124, *f*^2^ = 1.00]. However, there were no differences in reaction times for non-manipulated items across all valence-operation pairs [*F*_(3, 348)_ = 1.79, *p* = 0.15, $\eta_{p}^{2}$ = 0.02; BF = 0.13, *f*^2^ = 0.15]. Therefore, reaction times for non-manipulated items were collapsed across conditions to obtain a common baseline that is used for comparisons in all subsequent analyses. Maintain cues led to faster reaction times for both negative [*t*_(87)_ = −5.99, *p* < 0.001, *d* = 0.55; BF = 267,555.90, *r* = 0.71] and positive [*t*_(87)_ = −3.56, *p* < 0.001, *d* = 0.38; BF = 36.51, *r* = 0.71] items, compared to the non-manipulated baseline. Suppress cues lead to slower reaction times for both negative [*t*_(87)_ = 4.26, *p* < 0.001, *d* = 0.30; BF = 361.36, *r* = 0.71] and positive [*t*_(87)_ = 4.50, *p* < 0.001, *d* = 0.36; BF = 810.33, *r* = 0.71] items, compared to the non-manipulated baseline. There was no effect of valence on reaction times for either operation [Maintain: *F*_(1, 87)_ = 0.22, *p* = 0.64, $\eta_{p}^{2}$ = 0.003, BF = 0.12, *f*^2^ = 0.14; Suppress: *F*_(1, 87)_ = 0.13, *p* = 0.72, $\eta_{p}^{2}$ = 0.001, BF = 0.12, *f*^2^ = 0.14].

## Discussion

The current study aimed to investigate if maintenance suppression reduces access to emotional information in working memory as it does for neutral information. We found that it does so regardless of valence. We utilized complex, emotional scene images that were standardized based on normative ratings of valence and arousal from a separate sample of individuals. Two images of the same valence were designated to be encoded on each trial, with valence being interleaved across each run. We found that individuals were indeed slower to recognize items that were cued for suppression relative to uncued items, regardless of whether their normative valence was negative or positive. Likewise, individuals were faster to recognize items that were cued for maintenance relative to uncued items, regardless of valence. These results replicate and extend our prior study that reported a slowdown for suppressed items and a speedup for maintained items when the stimuli were emotionally neutral, and they suggest that individuals can reduce access to salient emotional information in working memory by engaging cognitive control. While prior research utilizing other paradigms has shown mixed results regarding the effect of emotion on working memory, this is the first study examining whether emotion influences processes that work to specifically remove information from working memory (as compared to gating it into working memory or shifting attention to other information in working memory). While one might wonder how to interpret our null effect of valence on maintenance suppression, it should be noted that we did observe some effects of valence in other measures in our study. Most notably, we found that accuracy for correct recognition of non-maintained items was higher for negative than positive stimuli, consistent with the suggestion that negative information does receive priority in processing, at least when it is not the focus of cognitive control.

Previous research investigating the effects of emotion on memory control have found varying results across paradigms. Prior work investigating the role of emotion on retrieval suppression for long-term memory in Think/No-Think (TNT) demonstrated that both negative and neutral information could be forgotten under explicit retrieval suppression instructions (van Schie et al., 2013), and in some instances that this effect was exaggerated for negative information (Depue et al., 2006). Other long-term memory forgetting research using item-method directed forgetting paradigms has shown that negative to-be-forgotten items are better remembered than neutral to-be-forgotten items (for meta-analysis, see Hall et al., 2021). The discrepancy between these two literatures may be due to the lack of an explicit strategy in directed forgetting studies. Whereas, maintenance suppression and retrieval suppression provide a direct instruction to participants, directed forgetting studies typically provide the vague instruction to individuals that “items followed by a remember cue will be tested later and items followed a forget cue will not be tested and you should do your best to forget them” (Wang et al., 2019). Including specific instructions may allow individuals to more effectively direct cognitive resources necessary to control salient information in memory, while more vague instructions likely lead to a variety of strategies used across individuals that may not be as effective at keeping intrusive information at bay.

Additionally, we did not observe any effects of emotional valence for items that were cued to be maintained. The absence of a valence effect during active maintenance in the current study may be because a single cued item is presumably held in an individual's focus of attention (Oberauer, 2002) with a heightened state of accessibility. The increased salience of a negative picture may not enhance its accessibility any further since it is already prioritized in the focus of attention. In line with this rationale, prior research has shown that both negative and positive information can have a facilitating effect on working memory maintenance compared to neutral items (Lindström and Bohlin, 2011). The results from the aforementioned work support our finding that emotional information deliberately maintained in working memory is faster responded to, regardless of valence, compared to unmaintained information.

### Limitations

There are some important limitations to this study that could be addressed in follow-up experiments, the first being that a non-clinical sample was tested in the current study. We did not select individuals with a history of repetitive negative thought, nor did we include any post-experiment questionnaires to assess individual differences that may alter the current results. While we did not find an effect of valence in this non-clinical sample, future work should examine these processes in a clinical sample that is more affected by negative repetitive thinking as emotional valence might have a more potent effect in such individuals.

Another limitation of the current design is we did not include non-emotional stimuli as a potential baseline, and hence cannot say whether there is a general effect of emotional information on maintenance suppression. While most research that investigates the effects of emotion on memory process typically include neutral stimuli to interpret whether emotion enhances or impairs performance compared to non-emotional information (e.g., Kensinger and Corkin, 2003; van Schie et al., 2013), our focus here was on the specific effect of negative vs. positive valence and hence a neutral baseline was not included.

An additional limitation of this study is that only image valence was considered as a factor in our analyses, while most models of emotion consider it to be a multidimensional construct that includes dimensions of valence and arousal (Russell, 1980) that jointly influence emotion's memory-modulating effect (e.g., Bergmann et al., 2012; Kensinger and Corkin, 2004; Mather et al., 2006). In the current study, the majority of negative images were rated as highly arousing, and the majority of positive images were rated as lowly arousing (see Methods). The fact that we did not find an effect of an item's valence, despite negative items having higher arousal ratings compared to positive items, suggests that, at least within our stimulus set, valence is not an important factor. Perhaps with more extreme stimuli, arousal could arise as a factor.

Another possible limitation of the current design is that a single category of images (i.e., scenes) were used throughout the experiment. Future work should explore the potential effects of other categories on intentional removal processes. For example, previous research has shown that face stimuli may hold a privileged status in memory, as indexed by increased recognition memory across various time intervals (Sato and Yoshikawa, 2013) and increased functional connectivity with brain regions involved in attention (Lin et al., 2019). Given these differences in processing, it is possible that reducing access to emotional faces may be more challenging due to their prioritized status in memory.

### Future directions

There are a variety of ways in which the current work could be expanded to provide a fuller picture of the effect of valence on maintenance suppression. One possible route to manipulate emotion more effectively, while still using images, is to tailor the stimuli to each participant. Recent work has taken this approach by asking participants to generate negative (i.e., “fears”), neutral, and positive (i.e., “hopes”) future events to use as stimuli in the main experiment (Mamat and Anderson, 2023). This may be a more ecologically valid approach because individuals are attempting to control autobiographical information, rather than arbitrary and subjective images.

Another potential way to manipulate emotion, without using inherently emotional images, is through Pavlovian fear conditioning, in which a previously neutral stimulus is paired to an aversive outcome and leads to a conditioned fear response (for review, see LeDoux, 2000). A recent study took this approach in an item-method directed forgetting paradigm and found that individuals showed worse memory for items paired with electric shocks that were instructed to be forgotten compared to ones instructed to be remembered, as well as diminished physiological responses to the to-be-forgotten items that were paired with shocks (Chalkia et al., 2023).

Another possible way to manipulate emotion could adapt a robust phenomenon from social psychology, mnemic neglect. The mnemic neglect model suggests that individuals are motivated to engage self-protection mechanisms. In experimental paradigms utilizing the mnemic neglect model, individuals are typically asked to encode personality traits about the self and are later given a memory test. Individuals show increased memory for low self-threatening (i.e., positive) traits and decreased memory for high self-threatening (i.e., negative) traits (Rigney et al., 2021; Sedikides and Green, 2006, 2009). This method of operationalizing emotion may be fruitful since some psychiatric disorders (e.g., depression) are characterized by repetitive negative thoughts about the self. Future research should explore using other forms of emotional stimuli in a maintenance suppression paradigm, as well as how emotion influences the neural correlates of maintenance suppression.

Another important future direction is to address individual differences in the ability to exert control over information currently occupying working memory. These differences could exist at the subject level (e.g., mental health, emotional states) or at the stimulus level (e.g., valence, task relevance, task content vs. task context), and may influence how control is applied to working memory processes (for meta-anlyses, see Schweizer et al., 2019; Xie et al., 2023). Future research should explore how these individual differences affect working memory, as well as how emotion shapes both the content and processes involved in working memory.

## Conclusion

In conclusion, our study is the first to investigate the role of emotion on maintenance suppression in working memory. Specifically, we demonstrate that maintenance suppression can reduce access to information with either positive or negative valence. Overall, these findings provide a better understanding of how healthy individuals can exert control over salient information and suggests a potential strategy to regulate negative, intrusive thoughts.

## Data Availability

The raw data supporting the conclusions of this article will be made available by the authors, without undue reservation.
